# Effects of a multi-strain *Bacillus subtilis*-based direct-fed microbial on weanling pig growth performance and nutrient digestibility

**DOI:** 10.1093/tas/txab058

**Published:** 2021-03-20

**Authors:** Jaron R Lewton, Adrienne D Woodward, Ronny L Moser, Kyan M Thelen, Adam J Moeser, Nathalie L Trottier, Robert J Tempelman, Dale W Rozeboom

**Affiliations:** 1 Department of Animal Science, Michigan State University, East Lansing, MI 48824, USA; 2 United Animal Health, Sheridan, IN 46069, USA; 3 Department of Large Animal Clinical Sciences, College of Veterinary Medicine, Michigan State University, East Lansing, MI 48824, USA

**Keywords:** amino acid, digestibility, direct-fed microbial, weanling pig

## Abstract

A study was conducted to evaluate the effects of a multi-strain *Bacillus subtilis*-based direct-fed microbial (DFM) on growth performance and apparent nutrient digestibility of nursery pigs. Eighty pigs, of equal number of barrows and gilts (initial body weight: 7.0 ± 0.60 kg), were weaned at 21 ± 1 d and randomly allotted to 1 of the 16 pens, with 5 pigs per pen. Two dietary treatments were implemented, a basal control (CON) and a control plus DFM (CDFM). Both diets were corn, soybean meal, and distillers dried grains based. Diets were fed for 42 d and growth performance measures were recorded weekly. On days 21 and 42 of the experiment, one pig per pen, with equal number of males and females, was randomly selected and euthanized. Digestibility of nitrogen (N), amino acids (AA), and energy were evaluated within the duodenum, jejunum, ileum, and ascending and distal colon. Relative to CON, CDFM tended to increase ADG during week 2 (*P =* 0.08) and significantly increased ADFI during week 2 (*P =* 0.04) and week 3 (*P =* 0.02). In addition, CDFM decreased the gain to feed ratio (G:F) during week 6 relative to CON (*P =* 0.04). Within the jejunum, pigs fed the DFM had greater digestibility of tryptophan (*P =* 0.04) and cysteine (*P =* 0.04) and tended to have greater digestibility of lysine (*P =* 0.07), methionine (*P =* 0.06), and threonine (*P =* 0.08), relative to CON. The content pH in the ascending colon did not differ between CDFM and CON. Compared with CON, apparent total tract digestibility (ATTD) of energy did not differ from CDFM, whereas ATTD of nitrogen of CDFM was lower (*P =* 0.05). The addition of a multi-strain *B. subtilis*-based DFM appears to impact growth performance, AA, and N digestibility depending upon the location in the gastrointestinal tract, with primary AA differences occurring within the mid-jejunum.

## INTRODUCTION

With the concerns regarding antibiotic-resistant bacteria reducing antibiotic effectiveness in humans, the use of antibiotics in swine is increasingly regulated (Aarestrup et al., 2010; Schultz and Rademacher, 2017). Alternative ways to achieve similar health and performance include the use of direct-fed microbials (DFM’s; Chen et al., 2005). Feeding a DFM composed of *Bacillus subtilis* improves pig growth performance (Kim et al., 2019) and nutrient digestibility (Lee et al., 2014; Blavi et al., 2018). Strains within the *B. subtilis* species have considerable genomic diversity, which imparts a range of strain-specific capabilities, likely contributing to the ability of this species to inhabit a myriad of terrestrial and aquatic environments including the mammalian gastrointestinal tract (GIT; Earl et al., 2008). A two-strain *B. subtilis* combination comprised of strains isolated from intestinal epithelial scrapings of high-performing pigs showed promising performance benefits for nursery pigs, increasing gain by 5% to 10% and lowering feed/gain ratio up to 5% (Augspurger et al., 2016); however, the mechanisms for improved growth were not elucidated. Given the gastrointestinal origin, it was hypothesized that improved digestibility of nutrients may be one mechanism by which the multi-strain combination may provide benefit. Moreover, although there are several previous studies involving either a single-strain *B. subtilis* or multiple strains within the *Bacillus* genus, there are no published studies involving multiple strains of this specific species. Therefore, the specific objective of this study was to evaluate the effect of a multi-strain *B. subtilis*-based DFM on growth performance and specific nutrient digestibility of the 21-d-old weanling pig. The hypothesis was that pigs fed a diet supplemented with *B. subtilis* have improved growth performance and greater digestibility of nitrogen (N), amino acids (AA), and gross energy (GE) within the different segments of the GIT.

## MATERIALS AND METHODS

### Animals, Housing, and Experimental Design

The Institutional Animal Care and Use Committee at Michigan State University reviewed and approved the protocol (PROTO201900154) for this experiment. The animal study was structured as a completely randomized design and conducted between the months of August and September 2019 at the Michigan State University Swine Teaching and Research Center. Eighty crossbred pigs (PIC 359 × Yorkshire) equally balanced by sex. Pigs were weaned at 21 ± 1 d (7.0 ± 0.6 kg, initial body weight) and randomly allotted into 16 cohorts with five pigs per cohort. Cohorts were randomly allotted to 1 of the 16 pens (1.22 × 1.83 m) located in 1 of the 4 mechanically ventilated identical nursery rooms. Cohort allotment was based on litter (dam), weight, and sex, and maintaining a similar average weight in each pen. Treatments were randomly assigned to each pen. Each pen held five pigs with four of the pens within each treatment containing three gilts and two barrows and the remaining four pens containing three barrows and two gilts. Pens were equipped with round-rod steel flooring, vertical-rod, fiberglass fencing and gates, single-sided two-hole feeders, and one nipple drinker. Pen to pen cross-contamination between the two treatments was considered minimal as pens and alleyways were cleaned on a regular basis. Rooms were operated on an all-in/all-out system and were disinfected using bleach (15.6 mL/L) 2 to 5 d before pigs were placed in the pens. All dams were vaccinated pre-breeding for parvovirus, leptospirosis, and erysipelas. Processing of newborn pigs on days 1 and 2 included ear notching, tail docking, and 1.0 mL iron dextran (200 mg/mL). All pigs received an additional 1.0 mL iron dextran (200 mg/mL) between days 7 and 10, and males were castrated. Pigs were vaccinated at weaning for the prevention of circovirus and erysipelas. All water nipples had a flow rate of 25 ± 1 mL/s.

### Diets and Feeding

Two dietary treatments were used: a control diet with no DFM supplementation (CON) and diet with supplementation of a multi-strain *B. subtilis*-based DFM (CDFM) (United Animal Health, Sheridan, IN) comprised of a dried spore preparation having a guaranteed count of 1.48 × 10^8^ CFU/g and included at a rate of 0.5 g/kg of feed to provide a final count of at least 7.35 × 10^4^ CFU/g of complete feed. Treatments were imposed over three dietary phases (days 0 to 14, 14 to 28, and 28 to 42) with day 0 representing the day of weaning (21 ± 1 d of age). All diets (Tables 1 and 2) were based on requirements published by the NRC (2012) and formulated according to example diets made available online by Kansas State University (Menegat et al., 2019). Dietary Cu and Zn were maintained at requirement (NRC, 2012). An indigestible marker, Titanium (Ti), was included in phases two and three in the form of titanium dioxide, at an inclusion rate of 0.1% of the complete diet. Diets were mixed at the Michigan State University swine farm using a 113-kg paddle ribbon mixer. The mixer was emptied and wiped clean between each batch to minimize cross-contamination. Analyzed feed values were obtained for each dietary phase, from composite samples of individual feeders from the same treatment (Table 3).

**Table 1.** Composition of diets, CON and diet containing a multi-strain *Bacillus subtilis*-based DFM (CDFM), across all three dietary phases, as-fed basis^†,‡^

Ingredient %	Phase 1, day 0 to 14	Phase 2, day 14 to 28	Phase 3, day 28 to 42
Corn	40.18	48.25	50.89
Soybean meal, 47.5% CP	17.30	21.15	22.65
Corn DDGS, 7.5% oil	5.00	10.00	20.00
Dried whey, 72% lactose	25.00	10.00	—
Fish meal	3.00	4.50	—
Spray-dried bovine plasma	4.00	—	—
Corn oil	3.00	3.00	3.00
Calcium carbonate, 38.5% Ca	0.65	0.60	0.85
Monocalcium phosphate, 21.5% P	0.55	0.55	0.45
Salt	0.30	0.55	0.60
l-Lysine HCl	0.35	0.50	0.65
dl-Methionine	0.15	0.15	0.14
l-Threonine	0.13	0.19	0.21
l-Tryptophan	0.03	0.06	0.06
l-Valine	0.07	0.10	0.10
VTM premix^||^	0.25	0.25	0.25
Phytase	0.05	0.05	0.05
Titanium dioxide^$^	—	0.10	0.10
Total, 100%	100	100	100

^†^CON and CDFM diets differed only by the inclusion of DFM at 0.05 g/kg or 1.48 × 108 CFU/g of complete feed.

^‡^CDFM (United Animal Health, Sheridan, IN) had a guaranteed count of 1.48 × 108 CFU/g and was included at a rate of 0.5 g/kg of feed to provide a final count of at least 7.35 × 104 CFU/g of complete feed.

^||^Vitamin Trace Mineral (VTM) premix provided the following vitamin and micromineral concentrations per kilogram of premix: Zinc 83.4 g, iron 66.7 g, manganese 33.4 g, copper 10 g, iodine 0.3 g, selenium 0.2 g, vitamin A 7,363 KIU, vitamin D 1,177 KIU, vitamin E 44,112 IU, menadione 1.5 g, vitamin B12 0.02 g, riboflavin 4.7 g, pantothenic acid 14.7 g, niacin 29.4 g, thiamine 0.7 g, pyridoxine 2.9 g, folic acid 1.1 g, and biotin 0.1 g.

^$^Titanium dioxide was included as an indigestible marker in phases two and three at 0.1% of the diet.

**Table 2.** Calculated analysis of diets, CON and diet containing a multi-strain *Bacillus subtilis*-based DFM (CDFM), across all three dietary phases, as-fed basis^†,‡^

Item	Phase 1, day 0 to 14	Phase 2, day 14 to 28	Phase 3, day 28 to 42
ME, kcal/kg^||^	3,477	3,439	3,417
CP, %	21.40	21.70	21.60
Lys SID, %^||^	1.40	1.35	1.30
His SID, %	0.48	0.47	0.48
Ile SID, %	0.77	0.76	0.70
Leu SID, %	1.65	1.62	1.69
Met + Cys SID, %	0.78	0.76	0.73
Thr, SID, %	0.88	0.85	0.82
Trp SID, %	0.27	0.26	0.25
Val SID, %	0.97	0.93	0.90
Ca, %	0.78	0.74	0.59
STTD P, %^||^	0.63	0.56	0.43
Ca;P, ratio	1.12	1.11	1.09
Phytase, FTU/kg	257.5	257.5	257.5
Na, %	0.51	0.38	0.31
Cl, %	0.69	0.62	0.51

^†^CON and CDFM diets differed only by the inclusion of DFM at 0.05 g/kg or 1.48 × 108 CFU/g of complete feed.

^‡^CDFM (United Animal Health, Sheridan, IN) had a guaranteed count of 1.48 × 108 CFU/g and was included at a rate of 0.5 g/kg of feed to provide a final count of at least 7.35 × 104 CFU/g of complete feed.

^||^Diets were calculated based on Metabolizable energy (ME), standardized ileal digestible (SID) amino acids, and standardized total tract digestible (STTD) phosphorus.

**Table 3.** Analyzed composition of CON diet and diet containing a multi-strain *Bacillus subtilis*-based DFM (CDFM)^†^, across all three dietary phases

	Phase 1, day 0 to 14		Phase 2, day 14 to 28		Phase 3, day 28 to 42	
Item	CON	CDFM	CON	CDFM	CON	CDFM
DM, %	91.45	91.40	90.31	90.34	90.61	90.91
GE, kcal/kg	4,608	4,611	4,678	4,597	4,762	4,736
CP, %	21.70	21.40	21.70	22.00	21.90	22.60
Crude fat, %	4.50	4.30	5.20	5.10	6.60	6.70
Crude fiber, %	1.60	1.70	2.10	2.20	3.30	3.00
NDF, %	6.20	6.20	8.30	8.10	11.10	12.00
ADF, %	2.90	2.50	4.00	4.00	5.50	5.80
Titanium, ppm	—	—	684	671	661	692
Indispensable AA, %						
Arg	1.10	1.10	1.10	1.10	1.10	1.10
His	0.51	0.51	0.50	0.50	0.52	0.53
Ile	0.91	0.92	0.90	0.91	0.87	0.88
Leu	1.85	1.89	1.83	1.84	1.95	2.02
Lys	1.58	1.53	1.56	1.46	1.49	1.56
Met	0.50	0.42	0.45	0.48	0.42	0.46
Met + Cys	0.91	0.81	0.76	0.81	0.76	0.79
Phe	0.98	0.97	0.98	0.99	1.05	1.07
Thr	1.00	1.03	0.97	0.96	0.91	0.94
Trp	0.30	0.30	0.28	0.34	0.26	0.28
Val	1.13	1.15	1.08	1.10	1.05	1.07
Dispensable AA, %						
Ala	1.06	1.07	1.11	1.09	1.13	1.16
Asp	1.94	1.93	1.86	1.87	1.77	1.73
Cys	0.41	0.39	0.31	0.33	0.34	0.33
Glu	3.28	3.27	3.32	3.33	3.37	3.32
Gly	0.80	0.80	0.88	0.86	0.79	0.79
Pro	1.17	1.19	1.23	1.20	1.29	1.32
Ser	0.84	0.84	0.80	0.80	0.82	0.82
Tyr	0.66	0.68	0.65	0.63	0.68	0.68

^†^CDFM (United Animal Health, Sheridan, IN) had a guaranteed count of 1.48 × 108 CFU/g and was included at a rate of 0.5 g/kg of feed to provide a final count of at least 7.35 × 104 CFU/g of complete feed.

### Data Recording and Sample Collection

Weekly performance data were collected by weighing each pig individually each week to estimate pen average daily gain (ADG). Pen feed disappearance was measured by vacuuming out the remaining feed and subtracting that from total weekly feed additions to represent average daily feed intake (ADFI). Feed efficiency (gain to feed ratio [G:F]) was calculated by dividing pen ADG by the corresponding pen ADFI.

At the end of weeks 3 and 6, one pig per pen was humanely euthanized for the analysis of nutrient digestibility and pH of the ascending colon content. An equal number of males and females were euthanized from both treatments to leave four pigs per pen at the conclusion of week 3 and to maintain two of each sex per pen for the remainder of the study. One pig was euthanized at a time alternating between CON and CDFM. Pigs were sedated using a combination of Telazol (2.5 mg/kg), Ketamine (1.25 mg/kg), and Xylazine (1.25 mg/kg) in a single intramuscular injection with a 22-G needle. Pigs were then euthanized using sodium pentobarbital (1 mL/4.5 kg) in a single intracardiac injection with an 18-G needle.

Immediately after confirmation of death, pigs were opened lengthwise and the cecum was located. Immediately anterior to the cecum, the ileum was sealed off with string and cut along the mesentery for approximately 50 cm. Digesta anterior to this section and within the ileum was manually pushed into the 50 cm section before sealing off the proximal end and removing that section of the ileum. The ascending colon was then removed in a similar manner, sealing the proximal end, immediately distal to the cecum, and measuring a 50 cm section, then sealing the distal end and removing the whole segment of ascending colon, stretching it lengthwise by cutting along the mesentery. The distal colon segment was collected by sealing off both proximal and distal ends and removing the entire distal colon, from the beginning of the descending colon to the rectum. This section was about 20 cm in length. The jejunum was located by cutting along the mesentery for approximately 8 m proximal to the cecum to ensure the proper location of the mid-jejunum. Beginning at this location, a 30-m section of jejunum was removed after sealing both proximal and distal ends. Before sealing the proximal end, additional jejunal digesta was manually moved into this 30 cm section similar to that performed in the ileum. The duodenum was located by first locating the distal end of the stomach and sealing off the duodenum just distal to the gastroduodenal junction. Approximately 20 cm distal to this point, the duodenum was sealed for a second time before removing the entire section.

Upon removal of each individual segment, digesta was collected from each of the five sections (duodenum, jejunum, ileum, and ascending and distal colon) for GE, N, complete AA profile, and Ti analyses. All digesta samples were collected into labeled, plastic 50-mL tubes. Ileal, jejunal, and ascending colon digesta were collected into two separate tubes. Digesta was removed by cutting off one of the tied ends and gently stripping the digesta lengthwise from the tissue into each tube. Digesta from the duodenum and distal colon were limited and little to no digesta could be obtained from the duodenum. Only small amounts were collected from the distal colon from a limited number of animals. For those, the small amount was placed into a single tube for GE, N, and Ti analysis. After collection, each tubes cap was wrapped in paraffin paper and placed on ice, and then frozen at −20 °C until analysis.

After the removal of digesta from the ascending colon, the pH was immediately recorded using a pH reader equipped with a probe (Mettler Toledo, Columbus, OH).

### Chemical Analysis of Feed and Digesta

Feed samples were prepared as described below and then shipped to the University of Missouri Experimental Station Chemical Laboratory (Columbia, MO) for nutrient analysis (Table 3). The following analyses were performed: dry matter (DM), neutral detergent fiber (NDF), acid detergent fiber (ADF), hemicellulose, cellulose, lignin, and total dietary fiber, crude fiber, N, ether extract, individual AA, Ca and P, and ash. Neutral detergent fiber was determined by the use of neutral detergent and heat-stable amylase according to the methods of Van Soest et al. (1991). Hemicellulose was calculated as NDF − ADF. Titanium in the feed was calculated according to Myers et al. (2004). Individual AA were determined in accordance with the standard methods of AOAC (2006).

Samples of digesta were prepared for the analysis of GE, N, Ti, and individual AA. All samples were freeze-dried (HarvestRight 115V, 3/4HP Salt Lake City, UT). Immediately after being removed from the freezer, whole tube weights were recorded and tubes were thawed placing digesta in appropriately labeled weigh boats or whirl pack baggies to increase surface area. Samples were then refrozen before placing in the freeze drier. After complete drying of individual samples, samples were finely ground using a Willey mill micro grinder (Swedesboro, NJ) with a 1-mm mesh-sized screen.

Titanium in the digesta was analyzed using an adjusted protocol based on that of Myers et al. (2004). Samples were weighed (150 mg) into a 100 mL Digesdahl flasks. About 4 mL of concentrated H_2_SO_4_ was added to each flask, swirled to cover all digesta, and kept overnight to digest. Flasks were then placed on a Digesdahl burner (Model 23130-20, Loveland, CO) and vacuum system to boil the acid for 6 min, followed by the addition of 10 mL of 50% H_2_O_2_. After completely burning off H_2_O_2_, flasks were cooled and then diluted to the 100 mL mark with distilled water. Upon water dilution to the 100 mL mark, 160 µL of standards and individual samples were transferred to microplates in duplicate. Standard concentrations used for Ti analysis were 0, 0.5, 1.0, 1.5, and 2.0 mg/dL. Plates were read at an absorbance of 460 nm on a well plate reader (Molecular Devices SpectraMax Plus 384, San Jose, CA). All sample duplicates having a coefficient of variation < 5 were averaged for a final Ti concentration, while those exceeding coefficient of variation of 5 were analyzed a third time.

Due to the limited sample availability from the distal colon, N digestibility of all GIT segments was also analyzed on-site according to Hach et al. (1987) to confirm with the analyzed values from the jejunum, ileum, and ascending colon obtained by the University of Missouri. Upon confirmation, digesta values for N collected on-site were used in the final analysis when calculating the apparent digestibility.

Gross energy was analyzed using an Adiabatic Bomb Calorimeter (115VParr model 12141Parr Instrument Co., Moline, IL) according to the manufacturer’s instructions.

Individual AA were determined for digesta samples by the University of Missouri Experimental Station Chemical Laboratory (Columbia, MO) as described above. As the available sample was limited, proximate analysis of digesta was limited to N.

### Calculations and Statistical Analysis

Apparent digestibility was calculated for GE, N, and AA as follows:


% digestibility = [1 − (nutrient digesta / nutrient feed) * (marker feed / marker digesta)] * 100,

where marker represents analyzed Ti values in both the feed and digesta, and nutrient represents the analyzed value of individual nutrients in both the feed and digesta.

Data were analyzed using the PROC GLIMMIX procedure in SAS 9.4 (SAS Institute, Inc., Cary, NC) specifying pen as the experimental unit. Data were analyzed as repeated measures over time (week) for performance. Digestibility data were analyzed as double repeated measures over both time (week) and space (GIT segment). The model included the fixed effects of dietary treatment, week, and GIT segment and all possible two-way interactions. Pens nested within treatments were specified to be random effects. Compound symmetry with heterogeneous variances was used to account for greater variation in the ascending colon, compared with other GIT segments, and was found to be a better fitting model compared with a homogeneous variance specification. Because of this heterogeneous variance specification, all analyses incorporated the Kenward–Rogers adjusted degrees of freedom. Treatment means were separated using the Tukey–Kramer multiple comparison test. Differences were considered significant at *P <* 0.05 and tendencies at 0.05 *< P <* 0.10.

## RESULTS AND DISCUSSION

### Morbidity, Mortality, and Growth Performance

This study was designed to evaluate the effects of a multi-strain *B. subtilis*-based DFM on growth performance and nutrient digestibility. There was no mortality and only two pigs were treated, both belonging to the DFM treatment, one due to lameness and the other due to weight loss, for a total morbidity of 2.5%. These pigs were monitored and considered to have fully recovered and were gaining weight within a few days of treatment and, therefore, remained on test for the duration of the study. Overall, there were no differences in growth performance with means ± SD of 0.51 ± 0.05 kg/d, 0.79 ± 0.05 kg/d, and 0.66 ± 0.05 for ADG, ADFI, and feed efficiency (G:F), respectively. However, there were significant differences within individual weeks. Compared with CON, ADG tended to be greater during week 2 (*P =* 0.08), and ADFI was greater during weeks 2 (*P =* 0.04) and 3 (*P =* 0.02) for CDFM. Compared with CON, feed efficiency was lower during week 6 for CDFM (*P =* 0.04; Table 4). The study did not confirm performance results previously obtained by Augspurger et al. (2016), who observed a 5% to 10% greater gain and 1.4% to 5% greater feed conversion with the addition of the same multi-strain *B. subtilis*-based DFM. In the current study, with pen being defined as the experimental unit, eight pens were assigned to each treatment, as this was considered enough statistical power to detect differences in nutrient digestibility of 5% or 10% (Lee et al., 2014); however, this was not expected to provide enough power to mimic the differences in growth performance obtained by Augspurger et al. (2016). Furthermore, pen density, with five pigs per pen, favored maximum performance. Animals performed exceptionally well compared with other similar studies (Guo et al., 2006; Walsh et al., 2007; Lee et al., 2014; Tang et al., 2019).

**Table 4.** Weekly growth performance of CON diet and a multi-strain *Bacillus subtilis*-based DFM (CDFM)^†^

Item	CON	CDFM	SEM	*P-*value
ADG, kg				
Week 1	0.170	0.160	0.01	0.31
Week 2	0.295^y^	0.341^x^	0.02	0.08
Week 3	0.428	0.446	0.02	0.54
Week 4	0.581	0.573	0.03	0.82
Week 5	0.722	0.751	0.02	0.35
Week 6	0.832	0.809	0.03	0.59
Overall	0.507	0.513	0.01	0.67
ADFI, kg				
Week 1	0.274	0.260	0.01	0.25
Week 2	0.399^b^	0.436^a^	0.01	0.04
Week 3	0.630^b^	0.693^a^	0.02	0.02
Week 4	0.854	0.877	0.02	0.49
Week 5	1.146	1.165	0.03	0.63
Week 6	1.359	1.401	0.04	0.41
Overall	0.777	0.805	0.02	0.23
G:F				
Week 1	0.619	0.618	0.02	0.98
Week 2	0.735	0.779	0.03	0.25
Week 3	0.680	0.642	0.02	0.28
Week 4	0.679	0.653	0.02	0.28
Week 5	0.630	0.646	0.01	0.44
Week 6	0.613^a^	0.577^b^	0.01	0.04
Overall	0.659	0.653	0.01	0.58

^†^CDFM (United Animal Health, Sheridan, IN), performance data taken from *n* = 8 pens per treatment, five pigs per pen from day 0 to 21, and four pigs per pen from day 21 to 42.

^a,b^Values in a common row lacking a common superscript differ (*P* ≤ 0.05).

^x,y^Values in a common row lacking a common superscript tend to differ (*P* ≤ 0.10).

### Amino Acid Digestibility

Differences between treatments were found within the jejunum only (Tables 5 and 6). Because of insufficient amounts of digesta from the duodenum and distal colon, differences in AA digestibility between treatments for those segments could not be assessed. Amino acid digestibility in the large intestinal segments was determined as a proxy to microbial N metabolism. While there is little evidence for AA absorption across the large intestine of the pig, significant lysine transport across the apical membrane of the proximal colon was reported in the growing pig (Woodward et al., 2012). Thus, AA digestibility and, therefore, disappearance from the large intestine may also indicate in situ AA utilization by the colonocytes rather than complete absorption. Although the role of the hindgut in the global AA and N metabolism of the pig has been given little attention, its relevance in the context of the microbiota and the microbiome cannot be ignored. Therefore, AA and N digestibility values in segments of the large intestine were also evaluated. In the jejunum, compared with CON, digestibility was greater for tryptophan (11%, *P =* 0.04) and cysteine (17%, *P =* 0.04) and tended to be greater for lysine (*P =* 0.07), methionine (*P =* 0.06), and threonine (*P =* 0.08; Table 5). These results indicate the potential activity of *B. subtilis* by the middle of the small intestine, specifically the jejunum.

**Table 5.** Apparent jejunal digestibility of indispensable and dispensable AA, for CON diet and a multi-strain *Bacillus subtilis*-based DFM (CDFM)^†^

Item	CON	CDFM	SEM	*P-*value
Indispensable AA, %				
Arg	57.00	64.96	3.64	0.13
His	47.20	55.51	3.64	0.11
Ile	51.26	57.67	3.20	0.17
Leu	54.27	58.58	3.20	0.35
Lys	61.62^y^	70.61^x^	3.40	0.07
Met	67.09^y^	73.44^x^	2.22	0.06
Met + Cys	46.20^y^	55.31^x^	3.70	0.07
Phe	54.63	59.81	2.88	0.21
Thr	49.36^y^	58.47^x^	3.61	0.08
Trp	56.92^b^	68.02^a^	3.34	0.04
Val	49.77	57.63	3.56	0.13
Dispensable AA, %				
Ala	47.37	53.11	3.44	0.24
Asp	43.57	52.06	3.83	0.13
Cys	12.55^b^	29.41^a^	5.32	0.04
Glu	49.70	54.84	3.11	0.25
Gly	-8.69	-1.47	8.84	0.57
Pro	44.17	49.66	3.87	0.32
Ser	43.88	51.94	3.76	0.14
Tyr	51.59	58.13	3.37	0.18
All indispensable AA, %	57.89	62.31	3.17	0.31
All dispensable AA, %	45.00	48.14	3.93	0.55
All AA, %	47.87	54.44	3.43	0.18

^†^CDFM (United Animal Health, Sheridan, IN), digestibility coefficients within the jejunum, *n* = 6 to 8 representative pigs per treatment at both days 21 and 42, and *n* = 3 to 4 for Trp day 21 due to lack of sufficient sample collection.

^a,b^Values in a common row lacking a common superscript differ (*P* ≤ 0.05).

^x,y^Values in a common row lacking a common superscript tend to differ (*P* ≤ 0.10).

**Table 6.** Apparent digestibility of indispensable AA, across segments of the GIT, for CON diet and a multi-strain *Bacillus subtilis*-based DFM (CDFM)^†^

Item %	CON	CDFM	SEM	*P*-value
Arg				
Jejunum	57.00	64.96	3.64	0.13
Ileum	74.80	76.27	3.34	0.76
Ascending colon	74.63	70.74	4.50	0.54
His				
Jejunum	47.20	55.51	3.64	0.11
Ileum	66.56	66.02	3.69	0.92
Ascending colon	69.21	65.60	4.93	0.60
Ile				
Jejunum	51.26	57.67	3.20	0.16
Ileum	67.87	68.27	3.66	0.94
Ascending colon	58.83	57.31	6.35	0.86
Leu				
Jejunum	54.27	58.58	3.20	0.35
Ileum	68.74	68.02	3.54	0.88
Ascending colon	65.95	62.23	5.27	0.61
Lys				
Jejunum	61.62^y^	70.61^x^	3.40	0.07
Ileum	73.28	75.99	3.35	0.57
Ascending colon	68.89	66.80	5.02	0.76
Met				
Jejunum	67.09^y^	73.44^x^	2.22	0.06
Ileum	76.23	78.43	2.64	0.55
Ascending colon	63.96	62.25	5.48	0.82
Phe				
Jejunum	54.63	59.81	2.88	0.21
Ileum	68.16	68.38	3.52	0.96
Ascending colon	65.16	61.04	5.36	0.58
Thr				
Jejunum	49.36^y^	58.47^x^	3.61	0.08
Ileum	62.50	62.31	4.10	0.97
Ascending colon	62.81	57.21	5.91	0.49
Trp				
Jejunum	56.92^b^	68.02^a^	3.34	0.04
Ileum	72.16	73.53	3.29	0.78
Ascending colon	76.94	76.12	3.58	0.87
Val				
Jejunum	49.77	57.63	3.56	0.13
Ileum	65.64	65.99	3.93	0.95
Ascending colon	57.78	56.46	6.54	0.88

^†^CDFM (United Animal Health, Sheridan, IN), all values are overall least square means of days 21 and 42 combined data, total AA were not reported in the distal colon due to insufficient sample size, *n* = 12 to 16 representative pigs per treatment, and *n* = 8 to 11 for Trp due to insufficient sample collection.

^a,b^Values in a common row lacking a common superscript differ (*P* ≤ 0.05).

^x,y^Values in a common row lacking a common superscript tend to differ (*P* ≤ 0.10).

Treatment differences in AA digestibility may have begun as early as the end of week 3 of the study. Compared with week 6, there was a consistent greater numerical difference between treatments during week 3 for the digestibility of nearly all indispensable AA, including arginine, histidine, isoleucine, lysine, methionine, threonine, tryptophan, and valine (Table 7). In some cases, the numerical difference in % digestibility between treatments was more than double at the end of week 3 compared with week 6. This was true for arginine (11.5% vs. 4.5%), histidine (12% vs 4.5%), and tryptophan (16.5% vs. 5.5%). These results indicate an early impact of the DFM on jejunal AA digestibility, at least within 3 wk of supplementation.

**Table 7.** Apparent digestibility of indispensable AA, by week within the jejunum, for CON diet and a multi-strain *Bacillus subtilis*-based DFM (CDFM)^†^

Item %	CON	CDFM	SEM	*P*-value
Arg				
Week 3	46.98	58.51	4.77	0.53
Week 6	67.01	71.41	4.24	0.98
His				
Week 3	34.62	46.37	5.20	0.60
Week 6	59.78	64.65	4.53	0.97
Ile				
Week 3	41.52	50.04	4.66	0.79
Week 6	61.00	65.31	4.07	0.97
Leu				
Week 3	44.22	49.18	4.69	0.97
Week 6	64.31	67.98	4.07	0.99
Lys				
Week 3	52.30	62.46	4.61	0.64
Week 6	70.94	78.75	4.03	0.74
Met				
Week 3	60.81	67.69	3.24	0.70
Week 6	73.36	79.19	2.82	0.69
Phe				
Week 3	43.79	50.67	4.19	0.85
Week 6	65.47	68.95	3.65	0.98
Thr				
Week 3	38.63	49.82	5.20	0.65
Week 6	60.09	67.11	4.53	0.88
Trp				
Week 3	47.07	63.53	5.28	0.35
Week 6	66.78	72.50	3.84	0.93
Val				
Week 3	40.64	50.28	5.17	0.77
Week 6	58.90	65.00	4.51	0.93

^†^CDFM (United Animal Health, Sheridan, IN), digestibility coefficients within jejunum, *n* = 6 to 8 representative pigs per treatment at both days 21 and 42, and *n* = 3 to 4 for Trp day 21 due to lack of sufficient sample collection.

^a,b^Values in a common row lacking a common superscript differ (*P* ≤ 0.05).

^x,y^Values in a common row lacking a common superscript tend to differ (*P* ≤ 0.10).

Isaacson and Kim (2012) showed differences in the microbiota within different GIT segments, with the jejunum being composed of primarily bacteria belonging to the Firmicutes phyla (> 90%), and the ileum composed of a mix of Firmicutes and Proteobacteria. Their findings agree with the effects of *B. subtilis* on digestibility within the jejunum in the current study. As a member of the Firmicutes phyla, *B. subtilis* may have helped restore the preferred microbiome of the jejunum, being a naturally Firmicutes-dominated environment. There are limited studies evaluating the effects of *B. subtilis* or other similar DFM’s on AA digestibility. Kaewtapee et al. (2017) recently evaluated AA digestibility of growing pigs with and without a mixed *Bacillus* spp. DFM containing one strain of both *B. subtilis* and *Bacillus licheniformis*. When fed with a wheat, barley, and soybean meal-based diet, DFM addition did not impact the digestibility of any AA by the terminal end of the ileum. Similarly, in the present study, no differences were observed in the ileal digestibility of AA.

*Bacillus subtilis* may impact the digestibility of AA and other nutrients through altered enzyme secretion including α-amylase, arbinase, cellulase, dextranase, lavansucrase, maltase, alkaline protease, neutral protease, and β-glucanase (Priest, 1977). Blavi et al. (2018) reported that increases in apparent total tract digestibility (ATTD) of energy may be due to the ability of *B. subtilis* to secrete α-amylase, which catalyzes the hydrolysis of glycosidic bonds in starch. They suggested that increases in energy utilization may be the result of increased digestion of fiber due to other enzymes secreted by *B. subtilis* including pectinase and xylanase. However, differences in GE digestibility were not observed in this study. Other research attributes changes in growth performance and digestibility to the proteases produced by *B. subtilis* (Tang et al., 2019) or unidentified enzymes that aid in digesting a variety of substrates present in soybean meal and other common swine feed ingredients (Giang et al., 2012; Upadhaya et al., 2015). The mechanisms of action for each single strain or mixed cocktail appear to differ. The DFM used in the current study appears to improve the digestibility of specific AA, rather than directly impacting energy digestibility. This may be an indication that the *B. subtilis* strains involved in this study secrete unique proteases, specifically improving absorption of cysteine and tryptophan, and potentially improving absorption of lysine, methionine, and threonine (Table 5). Recent research has been conducted to evaluate the dietary supplementation of proteases in nursery pig diets. Two separate studies conducted in 2016 (Pan et al., 2016; Yu et al., 2016) found improved apparent ileal digestibility (AID) of several AA both indispensable (arginine, histidine, isoleucine, leucine, lysine, methionine, and threonine) and dispensable (alanine, cysteine, and tyrosine) with protease supplementation. However, neither protease used in those two studies improved digestibility of tryptophan, the AA of which digestibility was affected by the DFM used in the current study.

The DFM used herein appeared to have a more pronounced impact on indispensable AA digestibility compared with dispensable AA (Table 5). Improvements in indispensable AA digestibility may be correlated with improved growth performance as demonstrated in previous research (Nortey et al., 2007; Min et al., 2009). Thus, greater utilization of many of the most limiting AA in corn-, soybean meal-, and distillers dried grains (DDGS)-based diets may have played an important role in the improvements in gain and feed efficiency observed in the studies conducted by Augspurger et al. (2016).

Regarding the AA digestibility in the large intestine, dietary supplementation of the DFM used in this study had no effect. We acknowledge that the extensive metabolism of AA by the microbiota, including utilization and synthesis, may have hampered finding distinct differences between AA digestibility; however, as discussed below, N digestibility was affected.

### Digestibility of GE and Nitrogen

Limited studies have determined the nutrient digestibility in multiple segments of the GIT. In the present study, digestibility of GE and N numerically increased from proximal to distal segments with CON digestibility values of 52.0%, 62.1%, 70.2%, and 77.6% for GE in the jejunum, ileum, ascending colon, and distal colon, respectively (Table 8). The same trend was found for N digestibility values for CON with 43.2%, 55.8%, 57.4%, and 71.3% for the jejunum, ileum, ascending colon, and distal colon, respectively (Table 8). While apparent nutrient digestibility values in small intestinal segments represent apparent nutrient absorption and post-gut availability to the animal, those of the large intestine are a consequence of microbial utilization and metabolism and indicative of urea-N recycling across the large intestinal wall. The values obtained from CON appear to be small, yet similar to those obtained from other weanling pig studies. Giang et al. (2012) fed weaned pigs a lactic acid bacteria supplement in addition to *B. subtilis* and observed greater digestibility values for N than the current study, with an AID of N approaching 80% and ATTD close to 85% to 90%. In a different study, ATTD was found to be close to 70% for N and 80% for GE, 4 wk into the nursery phase in pigs fed *B. subtilis* fermentation biomass (Lee et al., 2014). The ATTD of both N and GE fell between 70% and 80%, with a slight decrease in both measures from day 14 to 42 postweaning in pigs fed a multi-strain DFM composed of one strain of *B. subtilis* and two strains of *Bacillus amyloliquefaciens* (Cai et al., 2015). Tang et al. (2019) suggested that the *B. subtilis* may have a greater impact on digestibility in pigs fed low-protein diets. In their study, low protein combined with *B. subtilis* led to a 5% increase in ATTD of N (75% vs. 80%). Each of these studies observes a slight increase in either AID or ATTD with the addition of a *B. subtilis*-based DFM, which is in contrast with the results of the current study. These variations could be the result of differences in strains of bacteria, pig age and growth performance, dietary composition, inclusion rate, and/or interaction with other feed additives (Chesson, 1994; Chen et al., 2005). The diets fed in the current study were composed of corn, soybean meal, and DDGS. The inclusion of DDGS has been shown to significantly decrease the AID of GE and the ATTD of GE, N, and several essential AA (Agyekum et al., 2016) and may explain the relatively low digestibility values observed here.

**Table 8.** Apparent digestibility of GE, nitrogen, and total AA across segments of the GIT, for CON diet and a multi-strain *Bacillus subtilis*-based DFM (CDFM)^†^

Item, %	CON	CDFM	SEM	*P*-value
GE				
Jejunum	52.00	53.59	3.26	0.73
Ileum	62.13	60.00	3.77	0.69
Ascending colon	70.18	65.20	4.53	0.42
Distal colon	77.60	69.15	3.52	0.13
Nitrogen				
Jejunum	43.17	45.18	4.01	0.73
Ileum	55.77	59.21	4.53	0.59
Ascending colon	57.39	53.50	6.25	0.65
Distal colon	71.31^a^	58.91^b^	4.22	0.05
Total AA				
Jejunum	47.87	54.44	3.43	0.18
Ileum	65.01	64.96	3.85	0.99
Ascending colon	65.93	61.22	5.33	0.52

^†^CDFM (United Animal Health, Sheridan, IN), all values are overall least square means of days 21 and 42 combined data, total AA were not reported in the distal colon due to insufficient sample collection, and *n* = 6 to 8 representative pigs per treatment for both days 21 and 42.

^a,b^Values in a common row lacking a common superscript differ (*P* ≤ 0.05).

^x,y^Values in a common row lacking a common superscript tend to differ (*P* ≤ 0.10).

Relative to CON, overall N digestibility decreased (*P =* 0.05) in the distal colon with the addition of CDFM (71.3 vs. 58.9 ± 4.2 %; Table 8). Others reported greater fecal or ATTD of N when supplementing a *B. subtilis*-based DFM (Lee et al., 2014; Cai et al., 2015; Tang et al., 2019). The decrease in N digestibility may have been related to changes in the richness or diversity of the microbiome of the distal colon, leading to increased N recycling and endogenous secretions. Bacteria utilize and synthesize AA and other N-containing metabolites (Davila et al., 2013). Up to 25% of the urea produced in the liver enters the intestinal lumen, mostly not only within the small intestine but also in the colon in growing pigs (Bergen and Wu, 2009). Microbes are essential for the hydrolysis of urea into ammonia and carbon dioxide and subsequent conversion of ammonia to form glutamate and glutamine (Bergen and Wu, 2009). Glutamate digestibility was not measured within the distal colon due to limited sample volume. Glutamate digestibility values may have provided support in interpreting the difference in N digestibility in the distal colon.

### Colonic Contents pH

Agyekum et al. (2016) measured colonic pH as a secondary indication of changes in the microbiome or hindgut fermentation and production of volatile fatty acids (VFA). As the primary site of bacterial fermentation and microbial communities in nonruminants, we expected to see the greatest impact of *B. subtilis* on N digestibility, VFA production, and pH within the colon (Tajima and Aminov, 2015). However, no differences were observed in the pH or nutrient digestibility of the ascending colon content in this study. Over the course of the 42-d study, both treatments were fed increasing amounts of DDGS, 5%, 10%, and 20% for phases one, two, and three, respectively. Other studies have demonstrated the effects of DDGS on the microbiota of nursery pigs, finding that inclusion of 30% DDGS led to a significant decrease in the Firmicutes:Bacteroidetes ratio, mostly due to a decrease in many *Lactobacillus* species (Burrough et al., 2015). As they are known for producing lactic acid and reducing digest pH, a decrease in *Lactobacillus* within the ascending colon could result in an increase in digesta pH. However, this is not supported as pH was numerically decreased 5.8 vs. 5.6 in the control diet when increasing DDGS inclusion from 10% to 20% for pigs euthanized on weeks 3 and 6, respectively. Between weeks 3 and 6, colonic pH of CON and CDFM appeared to move in opposite directions, possibly indicating changes occurring within the microbiome with prolonged addition of *B. subtilis*. While *Lactobacillus* experiences optimal growth at a lower pH, *B. subtilis* has been shown to prefer an environment with a higher pH. Some studies suggest an optimal pH of 5.5, whereas others suggest a pH much closer to 6.5, depending on the specific strains and activity of interest (Chantawannakul et al., 2002; Koni et al., 2017).

In conclusion, supplementation of a multi-strain *B. subtilis*-based DFM appeared to have some beneficial effects on both growth performance and nutrient digestibility of nursery pigs. Digestibility was improved specifically within the jejunum, increasing the digestibility of cysteine and tryptophan, while tending to improve that of lysine, methionine, and threonine. These improvements in AA digestibility could help explain the improvements in ADG and G:F observed in previous studies conducted by Augspurger et al. (2016), which utilized the same DFM as the present study. *Bacillus subtilis* also appears to lead to changes in the hindgut digestibility and fermentation, observed through decreased digestibility of N within the distal colon relative to CON. The current study evaluated nutrient digestibility across multiple segments of the GIT. No previous studies feeding DFM’s have evaluated digestibility in the jejunum and ascending colon; thus, the data provided herein are novel information. While enzyme secretions related to protein digestion were not evaluated, we speculate that *B. subtilis* may be secreting enzymes that improve the digestibility of specific AA, which may in some cases impact the overall nitrogen digestibility. Other specific mechanisms relating to common indicators of pig health have been studied and reported elsewhere (Lewton et al., 2020). Additional studies are needed to identify specific mechanisms by which the multi-strain *B. subtilis*-based DFM may be improving AA digestibility in the jejunum and other GIT segments.

## References

[CIT0001] Aarestrup, F. M., V. F. Jensen, H. D. Emborg, E. Jacobsen, and H. C. Wegener. 2010. Changes in the use of antimicrobials and the effects on productivity of swine farms in Denmark. Am. J. Vet. Res. 71:726–733. doi:10.2460/ajvr.71.7.726. 20594073

[CIT0002] Agyekum, A. K., A. Regassa, E. Kiarie, and C. M. Nyachoti. 2016. Nutrient digestibility, digesta volatile fatty acids, and intestinal bacterial profile in growing pigs fed a distillers dried grains with solubles containing diet supplemented with a multi-enzyme cocktail. Anim. Feed Sci. Technol. 212:70–80. doi:10.1016/j.anifeedsci.2015.12.006.

[CIT0003] AOAC. 2006. Official methods of analysis. 18th ed. 2nd rev. ed. W. Hortwitz and G. W. Latimer Jr, editors. Ch. 45. AOAC International, Gaithersburg, MD.

[CIT0004] Augspurger, N. R., J. D. Spencer, S. Son, J. A. Ley, and M. R. King. 2016. Improved growth performance of nursery pigs fed diets supplemented with a *Bacillus subtilis*-based direct-fed microbial feed additive. J. Anim. Sci. 94(Suppl. 2):76. (Abstr.) doi:10.2527/msasas2016-162.

[CIT0006] Bergen, W. G., and G. Wu. 2009. Intestinal nitrogen recycling and utilization in health and disease. J. Nutr. 139:821–825. doi:10.3945/jn.109.104497. 19282369

[CIT0007] Blavi, L., J. N. Jorgensen, and H. H. Stein. 2018. Effects of *Bacillus amyloliquefaciens* and *Bacillus subtilis* on ileal digestibility of AA and total tract digestibility of CP and gross energy in diets fed to growing pigs. J. Anim. Sci. 97: 727–734. doi:10.1093/jas/sky432. PMC6358267 30445592

[CIT0008] Burrough, E. R., B. L. Arruda, J. F. Patience, and P. J. Plummer. 2015. Alterations in the colonic microbiota of pigs associated with feeding distillers dried grains with solubles. PLoS One. 10:e0141337. doi:10.1371/journal.pone.0141337. 26555787 PMC4640664

[CIT0009] Cai, L., S. Indrakumar, E. Kiarie, and I. H. Kim. 2015. Effects of a multi-strain *Bacillus* species-based direct-fed microbial on growth performance, nutrient digestibility, blood profile, and gut health in nursery pigs fed corn-soybean meal-based diets. J. Anim. Sci. 93:4336–4342. doi:10.2527/jas.2015-9056. 26440333

[CIT0010] Chantawannakul, P., A. Oncharoena, K. Klanbuta, E. Chukeatiroteb, and S. Lumyong. 2002. Characterization of proteases of *Bacillus subtilis* strain 38 isolated from traditionally fermented soybean in northern Thailand. Sci. Asia 28:241–245.

[CIT0011] Chen, Y. J., K. S. Son, B. J. Min, J. H. Cho, O. S. Kwon, and I. H. Kim. 2005. Effects of dietary probiotic on growth performance, nutrients digestibility, blood characteristics and fecal noxious gas content in growing pigs. Asian-Australas. J. Anim. Sci. 18:1464–1468. doi:10.5713/ajas.2005.1464.

[CIT0012] Chesson, A. 1994. Probiotics and other intestinal mediators. In: D. J. A. Cole, J. Wiseman, and M. A. Varley, editors, Principles of pig science. Nottingham Univ. Press, Loughborough, UK. p. 197–214.

[CIT0013] Davila, A. N., F. Blachier, M. Gotteland, M. Andriamihaja, P. H. Benetti, Y. Sanz, and D. Tome. 2013. Re-print of intestinal luminal nitrogen metabolism: role of the gut microbiota and consequences for the host. Pharm. Res. 69:114–126. doi:10.1016/j.phrs.2012.11.005. 23318949

[CIT0014] Earl, A. M., R. Losick, and R. Kolter. 2008. Ecology and genomics of *Bacillus subtilis*. Trends Microbiol. 16:269–275. doi:10.1016/j.tim.2008.03.004. 18467096 PMC2819312

[CIT0015] Giang, H. H., T. Q. Viet, B. Ogle, and J. E. Lindberg. 2012. Growth performance, digestibility, gut environment and health status in weaned piglets fed a diet supplemented with a complex of lactic acid bacteria alone or in combination with *Bacillus subtilis* and *Saccharomyces boulardii*. Livest. Sci. 143:132–141. doi:10.1016/j.livsci.2011.09.003.

[CIT0017] Guo, X., D. Li, W. Lu, X. Piao, and X. Chen. 2006. Screening of *Bacillus* strains as potential probiotics and subsequent confirmation of the in vivo effectiveness of *Bacillus subtilis* MA139 in pigs. Antonie Van Leeuwenhoek 90:139–146. doi:10.1007/s10482-006-9067-9. 16820971

[CIT0018] Hach, C. C., B. K. Bowden, A. B. Kopelove, and S. V. Brayton. 1987. More powerful peroxide Kjeldahl digestion method. J. AOAC Int. 70:783–787. doi:10.1093/jaoac/70.5.783.

[CIT0020] Isaacson, R., and H. B. Kim. 2012. The intestinal microbiome of the pig. Anim. Health Res. Rev. 13:100–109. doi:10.1017/S1466252312000084. 22853934

[CIT0022] Kaewtapee, C., K. Burbach, G. Tomforde, T. Hartinger, A. Camarinha-Silva, S. Heinritz, J. Seifert, M. Wiltafsky, R. Mosenthin, and P. Rosenfelder-Kuon. 2017. Effect of *Bacillus subtilis* and *Bacillus licheniformis* supplementation in diets with low- and high-protein content on ileal crude protein and amino acid digestibility and intestinal microbiota composition of growing pigs. J. Anim. Sci. Biotechnol. 8:37. doi:10.1186/s40104-017-0168-2. 28469845 PMC5410705

[CIT0023] Kim, K., Y. He., X. Xiong, A. Ehrlich, X. Li, H. Raybould, E. R. Atwill, E. A. Maga, J. Jorgensen, and Y. Liu. 2019. Dietary supplementation of *Bacillus subtilis* influenced intestinal health of weaned pigs experimentally infected with a pathogenic *E. coli*. J. Anim. Sci. Biotechnol. 10:52. doi:10.1186/s40104-019-0364-3.

[CIT0025] Koni, T. N. I., X. Rusman, C. Hanim, and X. Zuprizal. 2017. Effect of pH and temperature on *Bacillus subtilis* FNCC 0059 oxalate decarboxylase activity. Pak. J. Biol. Sci. 20:436–441. doi:10.3923/pjbs.2017.436.441. 30187731

[CIT0027] Lee, S. H., S. L. Ingale, J. S. Kim, K. H. Kim, A. Lokhande, E. K. Kim, and I. K. Kwon. 2014. Effects of dietary supplementation with *Bacillus subtilis* LS 1–2 fermentation biomass on growth performance, nutrient digestibility, cecal microbiota and intestinal morphology of weanling pig. Anim. Feed Sci. Technol. 188:102–110. doi:10.1016/j.anifeedsci.2013.12.001.

[CIT0028] Lewton, J. R., A. D. Woodward, R. L. Moser, K. M. Thelen, A. J. Moeser, N. L. Trottier, R. J. Tempelman, and D. W. Rozeboom. 2020. Effects of a multi-strain *Bacillus subtilis*-based direct-fed microbial on immunity markers, intestinal morphology, and microbial communities in diets fed to weanling pigs. J. Anim. Sci. 98(Suppl. 4):105–106. (Abstr.). doi:10.1093/jas/skaa278.193. PMC9278820 35854968

[CIT0031] Menegat, M. B., R. D. Goodband, J. M. DeRouchey, M. D. Tokach, J. C. Woodworth, and S. S. Dritz. 2019. Example of swine nursery diets. Kansas State University Swine Extension. Available from https://www.asi.k-state.edu/research-and-extension/swine/premix-and-diet-recommendations.html [accessed August 25, 2019].

[CIT0032] Min, B. J., J. H. Cho, Y. J. Chen, H. J. Kim, J. S. Yoo, Q. Wang, I. H. Kim, W. T. Cho, and S. S. Lee. 2009. Effects of replacing soy protein concentrate with fermented soy protein in starter diet on growth performance and ileal amino acid digestibility in weaned pigs. Asian-Australas. J. Anim. Sci. 22: 99–106. doi:10.5713/ajas.2009.70306.

[CIT0033] Myers, W. D., P. A. Ludden, V. Nayigihugu, and B. W. Hess. 2004. Technical Note: A procedure for the preparation and quantitative analysis of samples for titanium dioxide. J. Anim. Sci. 82:179–183. doi:10.2527/2004.821179x. 14753360

[CIT0035] Nortey, T. N., J. F. Patience, P. H. Simmins, N. L. Trottier, and R. T. Zijlstra. 2007. Effects of individual or combined xylanase and phytase supplementation on energy, amino acid, and phosphorus digestibility and growth performance of grower pigs fed wheat-based diets containing wheat millrun. J. Anim. Sci. 85:1432–1443. doi:10.2527/jas.2006-613. 17325125

[CIT0036] NRC. 2012. Nutrient requirements of swine. 11th rev. ed. Natl. Acad. Press, Washington, DC. p. 210–213.

[CIT0037] Pan, L., P. F. Zhao, Z. Y. Yang, S. F. Long, H. L. Wang, Q. Y. Tian, Y. T. Xu, X. Xu, Z. H. Zhang, and X. S. Piao. 2016. Effects of coated compound proteases on apparent total tract digestibility of nutrients and apparent ileal digestibility of amino acids for pigs. Asian-Australas. J. Anim. Sci. 29:1761–1767. doi:10.5713/ajas.16.0041 27004811 PMC5088425

[CIT0038] Priest, F. G. 1977. Extracellular enzyme synthesis in the genus *Bacillus*. Bacteriol. Rev. 41:711–753. 334155 10.1128/br.41.3.711-753.1977 PMC414021

[CIT0041] Schultz, L. L., and C. J. Rademacher. 2017. Food and drug administration guidance 209 and 213 and veterinary feed directive regulations regarding antibiotic use in livestock: a survery of preparation and anticipated impacts in the swine industry. J. Swine Health Prod. 25:247–255.

[CIT0044] Tajima, K., and R. Aminov. 2015. Structure and function of a nonruminant gut: a porcine model. In: A. K. Puniya, R. Singh, and D. N. Kamra, editors, Rumen microbiology: from evolution to revolution. Springer, New Delhi, India. p. 47–79.

[CIT0045] Tang, W., Y. Qian, B. Yu, T. Zhang, J. Gao, J. He, Z. Huang, P. Zheng, X. Mao, J. Luo, et al. 2019. Effects of *Bacillus subtilis* DSM32315 supplementation and dietary crude protein level on performance, gut barrier function and microbiota profile in weaned piglets. J. Anim. Sci. 97:2125–2138 doi:10.1093/jas/skz090. 30883644 PMC6488307

[CIT0046] Upadhaya, S. D., S. C. Kim, R. A. Valientes, and I. H. Kim. 2015. The effect of *Bacillus*-based feed additive on growth performance, nutrient digestibility, fecal gas emission, and pen cleanup characteristics of growing-finishing pigs. Asian-Australas. J. Anim. Sci. 28, 7: 999–1005. doi:10.5713/ajas.15.0066. 26104405 PMC4478510

[CIT0047] Van Soest, P. J., J. B. Robertson, and B. A. Lewis. 1991. Methods for dietary fiber, neutral detergent fiber, and nonstarch polysaccharides in relation to animal nutrition. J. Dairy Sci. 74:3583–3597. doi:10.3168/jds.S0022-0302(91)78551-2. 1660498

[CIT0049] Walsh, M. C., K. L. Saddoris, D. M. Sholly, R. B. Hinson, A. L. Sutton, T. J. Applegate, B. T. Richert, and J. S. Radcliffe. 2007. The effects of direct-fed microbials delivered through the feed and/or in a bolus at weaning on growth performance and gut health. Livest. Sci. 108:254–257. doi:10.1016/j.livsci.2007.01.051.

[CIT0050] Woodward, A. D., M. Z. Fan, R. J. Geor, L. J. McCutcheon, N. P. Taylor, and N. L. Trottier. 2012. Characterization of l-lysine transport across equine and porcine jejunal and colonic brush border membrane. J. Anim. Sci. 90:853–862. doi:10.2527/jas.2011-4210. 22064742

[CIT0052] Yu, G., D. Chen, B. Yu, J. He, P. Zheng, X. Mao, Z. Huang, J. Luo, Z. Zhang, and J. Yu. 2016. Coated protease increases ileal digestibility of protein and amino acids in weaned piglets. Anim. Feed Sci. Technol. 214:142–147. doi:10.1016/j.anifeedsci.2016.02.006.

